# Mumps Virus–associated Hemophagocytic Syndrome

**DOI:** 10.3201/eid1102.040993

**Published:** 2005-02

**Authors:** Kunihiko Hiraiwa, Katsuyuki Obara, Atsuhisa Sato

**Affiliations:** * Hamamatsu Red Cross Hospital, Hamamatsu, Japan;; † Mito Red Cross Hospital, Mito, Japan

**Keywords:** letter, mumps virus, histiocytosis, non-Langerhans-cell

**To the Editor:** Virus-associated hemophagocytic syndrome (VAHS) is a fulminant disorder associated with systemic viral infection and is characterized pathologically by the proliferation of hemophagocytic histiocytes in the lymphoreticular tissues. Here we report a case of mumps VAHS following parotitis and pancreatitis.

A 39-year-old, previously healthy woman sought treatment for abdominal pain on June 14, 2002. On physical examination, her bilateral parotid glands were swollen, and her left upper quadrant was tender. Laboratory studies showed a leukocyte count of 4,640/mm^3^, a hemoglobin concentration of 13.9 g/dL, and a platelet count of 19.1 x 10^4^/mm^3^. The level of amylase was elevated in her blood (1,613 IU/L; normal 50–160 IU/L) and urine (12,940 IU/L; normal 200–1,100 IU/L). Her level of pancreatic enzymes was also elevated: lipase level was 194 IU/L (normal 7–60 IU/L) and phospholipase A2 level was 1,340 ng/dL (normal 130–400 ng/dL). Parotitis and acute pancreatitis due to a mumps virus infection were diagnosed. After supportive therapy, the laboratory abnormalities improved.

On July 1, her temperature suddenly rose to 39°C. At that time, pancytopenia was evident, with a leukocyte count of 2,350/mm^3^, a hemoglobin concentration of 10.9 g/dL, and a platelet count of 9.1 x 10^4^/mm^3^. Laboratory studies showed an elevation of lactic dehydrogenase (1,403 IU/L; normal 180–460 IU/L), ferritin (12,727.0 ng/mL; normal 4.0–64.2 ng/mL), and soluble interleukin-2 receptors (1,660 U/mL; normal 145–519 U/mL). Hypercytokinemia was also shown, with an interleukin-6 of 12.7 pg/ml (normal <3.1 pg/ml). Her bone marrow was normocellular, and an increased number of histiocytes with hemophagocytosis was found. Extensive cultures and serologic studies for microbial and viral infections were all negative, whereas tests for immunoglobulin G and immunoglobulin M antibodies against the mumps virus were both positive. Mumps VAHS was diagnosed. Treatment with corticosteroids led to a complete remission of symptoms.

VAHS was initially reported by Risdall et al. in 1979 (*1*). Although the precise pathogenesis of VAHS remains unknown, current hypotheses focus on the roles played by activating cytokines. VAHS has been reported in connection with a variety of viruses: adenovirus, cytomegalovirus, dengue, Epstein-Barr, hepatitis A, hepatitis B, hepatitis C, herpes simplex, HIV, human herpesvirus 6, human herpesvirus 8, influenza A (antigenic type H1N1), measles, parainfluenza type III, parvovirus B 19, rubella, and varicella zoster (*2*). This report is the first of a VAHS case associated with a mumps virus infection. The clinical course of VAHS is highly variable, and in some cases, especially in Epstein-Barr virus infection, VAHS is a dramatic illness with a potentially fatal outcome (*2*). This case implies that mumps VAHS may have a positive prognosis.
